# The Hospital Problem

**Published:** 1920-09-25

**Authors:** 


### The Hospital Problem.

In the Quarterly Paper which they have just issued the Liverpool Council of Voluntary Aid deal with the present critical situation of the hospitals, and give an excellent survey of the problems and difficulties that have to be faced. They point out, to begin with, that there is no national system for the treatment of the sick, and the subject is therefore in strong contrast with certain other matters, such as education, relief of the poor, care of the mentally defective, etc., all of which are administered through a central Government department, with appropriate local statutory authorities. Instead we have (*a*) voluntary local nursing associations more or less completely covering the country; (*b*) voluntary hospitals possibly adequate in the large towns as a whole, but less so in the smaller towns and rural districts; (*c*) Poor-Law hospitals for the destitute sick; (*d*) infectious hospitals provided by the local health authorities; (*e*) medical inspection and treatment of school children by the local education authorities; and (*f*) National Health Insurance, for the medical treatment (excluding, in fact, nursing and hospital treatment) of insured persons.

The following points may be noted with regard to the above-mentioned bodies:—The first four are in no way officially connected with each other, though occasionally local working arrangements may be made in matters of detail. Neither the nursing associations nor the voluntary hospitals are linked up nationally, though attempts in this direction are being made (*e.g.*, by the British Hospitals Association) to federate the various hospitals as a whole. With regard to the voluntary hospitals in the larger towns, not even the local hospitals are in any organic relation, though in one or two towns representatives of the different institutions meet from time to time, and the Ministry of Health report contains a suggestion for a closer relation. The phrase "Destitute Sick" is loosely used, and some Poor-Law hospitals are definitely entering the sphere of the voluntary hospitals. In many cases Poor-Law hospitals have vacant bed accommodation, and in one or two cases, notably Bolton, paying wards have been introduced.

In actual practice, the function of the voluntary hospital is steadily tending to change from (*a*) an institution for the treatment of the poorer public in general to (*b*) an institution for the treatment of acute diseases and complicated operations demanding a highly skilled medical staff, elaborate apparatus, and a high rate of expenditure per bed. It would seem desirable, the Council say, from the point of view of economy, as well as efficiency, to reserve the expensive institutions for the expensive treatment. The following suggestions have been put forward by experienced persons to serve this end (*a*) the reviewing of local arrangements between the voluntary hospitals, or between the voluntary hospitals and the Poor-Law (or municipal) hospitals or to convalescent homes patients when the less acute and expensive cases; (*b*) the working out of a local scheme for evacuating to the less acute hospitals or to convalescent home patients when the acute stage is passed. It has been estimated that this would result in the number of cases passing through an acute hospital being increased by a third; (*c*) the extension of an interesting experiment recently adopted by one or two hospital surgeons of arranging in small towns for operations to be dealt with at the local cottage hospitals weekly or fortnightly.

The King Edward's Fund figures show that the 1918 expenses increased by 40 per cent. over the 1914 figures, and prices and wages have steadily risen since that date. It is an under estimate to say that speaking generally every voluntary hospital will have to increase its annual income by 50 per cent. to meet its annual expenses. Recent appeals show that it is not possible for this additional income to be secured from annual subscriptions. The sources which are being tried are (*a*) increase of the Hospital Saturday Fund; (*b*) employment of special officers at hospitals to secure more support from the patients; (*c*) making a definite charge towards the maintenance of in-patients (*e.g.*, London Hospital, £1 1s. per week); (*d*) introduction of more paying wards at hospitals—though this must often be done by reducing the accommodation for poorer patients. These methods are at times in the nature of an alternative—thus to what extent, it may be asked, can the contributions of employees to the Local Hospital Saturday Fund be raised and at the same time a definite charge for maintenance introduced. Reference is made to The Hospital for July 23, which outlined an ingenious system of voluntary insurance for hospital treatment, but the Council say, "it is a question how far this scheme is really feasible. Lastly there is the possibility of State or Rate Grants. In this connection it may be noted that the Government have stated that it is not their intention to take over the hospitals."

